# Mucolipidosis type III, a series of adult patients

**DOI:** 10.1007/s10545-018-0186-z

**Published:** 2018-04-27

**Authors:** Esmee Oussoren, David van Eerd, Elaine Murphy, Robin Lachmann, Jan C. van der Meijden, Lies H. Hoefsloot, Rob Verdijk, George J. G. Ruijter, Mario Maas, Carla E. M. Hollak, Janneke G. Langendonk, Ans T. van der Ploeg, Mirjam Langeveld

**Affiliations:** 1 Department of Pediatrics, Center for Lysosomal and Metabolic Diseases, Erasmus MC—Sophia Children’s Hospital, P.O. Box 2060, 3000 CB Rotterdam, The Netherlands; 2 Department of Internal Medicine, Center for Lysosomal and Metabolic Diseases, Erasmus MC, Rotterdam, The Netherlands; 3 Charles Dent Metabolic Unit, National Hospital for Neurology and Neurosurgery, Queen Square, London, UK; 4 Department of Clinical Genetics, Center for Lysosomal and Metabolic Diseases, Erasmus MC, Rotterdam, The Netherlands; 5 Department of Pathology, Erasmus MC, Rotterdam, The Netherlands; 6 Department of Radiology and Nuclear Medicine, Academic Medical Center, University of Amsterdam, Amsterdam, The Netherlands; 7 Department of Endocrinology and Metabolism, Academic Medical Center, University of Amsterdam, Amsterdam, The Netherlands

## Abstract

**Background:**

Mucolipidosis type III α/β or γ (MLIII) are rare autosomal recessive diseases, in which reduced activity of the enzyme UDP-*N*-acetyl glucosamine-1-phosphotransferase (GlcNAc-PTase) leads to intra-lysosomal accumulation of different substrates. Publications on the natural history of MLIII, especially the milder forms, are scarce. This study provides a detailed description of the disease characteristics and its natural course in adult patients with MLIII.

**Methods:**

In this retrospective chart study, the clinical, biochemical and molecular findings in adult patients with a confirmed diagnosis of MLIII from three treatment centres were collected.

**Results:**

Thirteen patients with MLIII were included in this study. Four patients (31%) were initially misdiagnosed with a type of mucopolysaccharidosis (MPS). Four patients (31%) had mild cognitive impairment. Six patients (46%) needed help with activities of daily living (ADL) or were wheelchair-dependent. All patients had dysostosis multiplex and progressive secondary osteoarthritis, characterised by cartilage destruction and bone lesions in multiple joints. All patients underwent multiple orthopaedic surgical interventions as early as the second or third decades of life, of which total hip replacement (THR) was the most common procedure (61% of patients). Carpal tunnel syndrome (CTS) was found in 12 patients (92%) and in eight patients (61%), CTS release was performed.

**Conclusions:**

Severe skeletal abnormalities, resulting from abnormal bone development and severe progressive osteoarthritis, are the hallmark of MLIII, necessitating surgical orthopaedic interventions early in life. Future therapies for this disease should focus on improving cartilage and bone quality, preventing skeletal complications and improving mobility.

**Electronic supplementary material:**

The online version of this article (10.1007/s10545-018-0186-z) contains supplementary material, which is available to authorized users.

## Introduction

Mucolipidoses type II/III α/β or γ (MLII OMIM#252500, MLIII α/β MIM#252600, MLIII γ OMIM#252605) are rare autosomal recessive diseases (Maroteaux and Lamy 1966; Leroy and Martin 1975; Raas-Rothschild et al. 2000, 2004; Cathey et al. 2008, 2010). In these conditions, activity of the membrane-bound hexameric enzyme UDP-*N*-acetyl glucosamine-1-phosphotransferase (GlcNAc-PTase), consisting of three subunits named α2, β2 and γ2, is absent or reduced (Reitman and Kornfeld 1981; Bao et al. 1996; Raas-Rothschild et al. 2000; Kudo et al. 2005; Tiede et al. 2005). The *GNPTAB* gene (chromosome 12q23.3; OMIM#607840) encodes for the α/β subunits and the *GNPTG* gene (chromosome 16; OMIM#607838) for the γ subunits. GlcNAc-PTase is responsible for the first step in the phosphorylation of enzyme-conjugated mannose residues to mannose-6-phosphate in the Golgi apparatus. Mannose-6-phosphate serves as the recognition marker, targeting newly synthesised lysosomal enzymes to the lysosome. In the absence or reduced presence of this marker, lysosomal enzymes are secreted in plasma, where they are unable to execute their function (Reitman and Kornfeld 1981), resulting in the accumulation of several substrates, such as glycosaminoglycans and (glyco)sphingolipids.

ML presents as a clinical spectrum. In the most severe form, MLII (OMIM#252500, I-cell disease), GlcNac-PTase activity is completely deficient, leading to severe and rapidly progressive airway, cardiac, skeletal and nervous system disease, resulting in death in early childhood (Leroy and Martin 1975; Cathey et al. 2008, 2010). MLIII α/β has a broader phenotypic range, from severely affected patients that die in childhood to milder affected patients displaying primarily skeletal symptoms, who survive into adulthood (Maroteaux and Lamy 1966; Bargal et al. 2006; Encarnação et al. 2009; Otomo et al. 2009; Cathey et al. 2010; David-Vizcarra et al. 2010; Yang et al. 2017). The patients with MLIII γ that have been described so far all have milder phenotypes (Raas-Rothschild et al. 2000, 2004; Falik-Zaccai et al. 2003; Persichetti et al. 2009; Liu et al. 2014; Tüysüz et al. 2018).

Clinical features that have been described in MLIII are mild coarsening of the face, corneal clouding, mild retinopathy, cardiac valve abnormalities, restrictive pulmonary function, tracheal/bronchial malacia, skeletal dysplasia, scoliosis, stiffness of the joints, short stature, claw hand deformity, carpal/tarsal tunnel syndrome and spinal cord compression (Haddad et al. 1997, 2000; Hetherington et al. 1999; Robinson et al. 2002; Raas-Rothschild et al. 2004; Steet et al. 2005; Cripe et al. 2009; Encarnação et al. 2009; Otomo et al. 2009; Smuts et al. 2009; Cathey et al. 2010; David-Vizcarra et al. 2010; Kerr et al. 2011; Kobayashi et al. 2011; Liu et al. 2014, 2016; Pantoja Zarza and Diez Morrondo 2014). Reports on intellectual performance and learning abilities vary from normal to mild cognitive impairment (Ward et al. 1993; Umehara et al. 1997; Raas-Rothschild et al. 2004; Tiede et al. 2005; Cathey et al. 2010; Kerr et al. 2011; Kobayashi et al. 2011; Cavalcante et al. 2012; Yang et al. 2017; Tüysüz et al. 2018). Publications on the natural history of adult MLIII patients are rare. Some single case studies or small case series of MLIII patients reaching adulthood have been published, but they lack systematic description of disease onset, progression over time and severity of the disease characteristics and surgical interventions (Raas-Rothschild et al. 2004; Encarnação et al. 2009; Otomo et al. 2009; Cathey et al. 2010; David-Vizcarra et al. 2010; Yang et al. 2017; Tüysüz et al. 2018).

Currently, there are no curative treatments for MLII and III. From experience in other extremely rare disorders (e.g. mucopolysaccharidosis, MPS) for which therapy became available, we recognise the importance of natural history data collection, especially of the milder cases, since the focus in the medical literature is often on the severe phenotypes. Once treatment becomes available, the latter may lead to an overestimation of treatment effect, as the course of the treated patients that are mildly affected is compared to severely affected patients reported in the literature. Natural history studies help to identify future therapeutic goals, aid counselling and provide the basis for tailored standardised follow-up of these patients. The aim of this study is to provide a detailed description of the disease characteristics of MLIII and its natural course, by studying data from adult patients.

## Methods

### Patients

In this retrospective medical record review, the clinical, biochemical and molecular findings from adult patients with a confirmed diagnosis of MLIII from three specialist centres were collected [the Academic Medical Center (AMC), Amsterdam, the Netherlands, Erasmus MC, Rotterdam, the Netherlands and the National Hospital for Neurology and Neurosurgery, London, United Kingdom).

The diagnosis of MLIII was established by the measurement of plasma and/or fibroblast activity of several lysosomal enzymes, including β-hexosaminidase A, β-hexosaminidase A + B, α-L-fucosidase, β-D-glucuronidase, α-D-mannosidase and β-D-galactosidase. In addition, in a subset of patients, GlcNAc-1-PTase activity in fibroblasts was measured or DNA analysis of the *GNPTAB* or *GNPTG* genes was performed.

Data on the demographic and general characteristics (age of initial/correct diagnosis and anthropometry), clinical symptoms, cognitive ability, highest education qualification, impairments in activities of daily living (ADL), wheelchair dependency, in employment, imaging results (radiographs and MRI scans), number and types of orthopaedic surgeries performed and echocardiography, pulmonary function tests, were collected from patient records.

## Results

### Patients’ characteristics

The characteristics of the 13 adult patients are outlined in Table 1. Median age at last follow-up was 30 years (range 18–68 years). Most patients were of Caucasian descent, and both genders were equally represented. Patients 9, 11 and 10, 12 are siblings. Out of the 13 patients, one was initially misdiagnosed as MPS II and three as MPS IV. In one of these patients, diagnosed with MPS IV at the age of 30 years, the correct diagnosis was established as late as age 64 years.

**Table 1:** Patient characteristics

Patient	Gender	Initial diagnosis (years)	Age at correct diagnosis (years)	Age at last follow-up (years)	Mutations of the *GNPTAB* gene		Mutations of the *GNPTG* gene		Multiple lysosomal enzyme activities	
					Allele 1	Allele 2	Allele 1	Allele 2	Plasma	Fibroblasts
1	M	MPS IV 8	11	23	NM_024312.4:c.1178A>G p.(His393Arg)	NM_024312.4:c.3503_3504del p.(Leu1168fs)[1]			Elevated	N.A.*
2	M		7	27	NM_024312.4:c.196C>T p.(Gln66*)	NM_024312.4:c.366-1G>C p.?[2]			N.A.	Reduced
3	F	MPS II 4	7	48			NM_032520.4:c.411 + 11_411 + 35del p.?	NM_032520.4:c.411 + 11_411 + 35del p.?	Elevated	Reduced
4	F		9	18	NM_024312.4:c.1090C>T p.(Arg364*)	NM_024312.4:c.2715 + 2T>G p.?			Elevated	N.A.
5	M		8	30			NM_032520.4:c.122_138del p.(Pro41fs)	NM_032520.4:c.331T>C p.(Trp111Arg)	Elevated	N.A.
6	M	MPS IV	46	48			NM_032520.4:c.318-1G>C p.?	NM_032520.4:c.318-1G>C p.?	N.A.	Reduced
7	F	MSP IVB 30	64	68			NM_032520.4:c.318-1G>C p.?	NM_032520.4:c.318-1G>C p.?	Elevated	N.A.
8	F		7	28	N.A.	N.A.	N.A.	N.A.	N.A.	Reduced
9^	M		22	28	N.A.	N.A.	N.A.	N.A.	Elevated	N.A.
10#	M		8	39	N.A.	N.A.	N.A.	N.A.	Elevated	N.A.
11^	F		17	32	N.A.	N.A.	N.A.	N.A.	Elevated	N.A.
12#	F		Unknown	35	N.A.	N.A.	N.A.	N.A.	Elevated	N.A.
13	F		3.5	27	N.A.	N.A.	N.A.	N.A.	Elevated	Reduced

*: GlcNAc-1-PT enzyme deficient in fibroblasts, N.A.: not available

#^; siblings

1. Kudo et al. (2005)

2. Raas-Rothschild et al. 2004

Most patients developed clinical symptoms in the first decade of life. The diagnosis of ML was made in ten patients by the establishment of elevated levels of lysosomal enzymes in plasma, confirmed by a concomitant decreased lysosomal enzyme activity in fibroblasts in four patients (in one patient, only enzyme measurements in fibroblasts were performed) and in one patient by a decreased activity of GlcNAc-1-PTase. In seven patients, DNA analysis was performed; three patients had mutations in the *GNPTAB* gene and four patients in the *GNPTG* gene. The mutations and clinical features of patient number 2 were published 13 years ago by Raas-Rotschild et al. (2004). The *GNPTAB* gene c.1178A>G; p.(His393Arg) mutation (patient number 1), the *GNPTG* gene homozygous variants c.411 + 9_411 + 35del27 (patient number 3) and c.318-1G>C (patient numbers 6 and 7) and the *GNPTG* gene heterozygous mutation c.122_138del; p.(Pro41fs) with the c.331T>C variant (patient number 5) have not been published before.

### Clinical signs and functioning in daily life

Five patients had notably short statures (range 129–158 cm, median 145 cm) (Table 2). In patient number 13, height could not be measured. Six patients needed help with ADL and/or were wheelchair-dependent. All but one patient suffered from carpal tunnel syndrome (CTS) (Supplemental Table 2).

**Table 2:** Anthropometry, cognitive involvement and functioning in daily life

Patient	Height (cm) and BMI (kg/m^2^) at last follow-up	Cognitive impairment (Y/N)	Highest education qualifications	Impairment in ADL/ wheelchair user (Y/N)	In employment (Y/N)
1	129 (16)	N	Secondary education	N/Y*	Y
2	171 (24)	N	Academic education	N/N	Y
3	144 (28)	N	Secondary education	N/N	y
4	170 (24)	Y, mild [IQ 65]	Secondary education	N/N	Y
5	170 (22)	Y, mild	Special needs education	Y/N	N#
6	176 (33)	N	Professional education	Y/N^	Y
7	150 (52)	N	N.A.	Y/Y (from age 23 years onwards)	Y
8	145 (24)	N	University	Y/Y	Y
9	179 (23)	N	University	N/N	Y
10	169 (29)	Y, mild [IQ 70]	Secondary school	N/N	N
11	158 (21)	N	College	N/N	Y
12	160 (20)	N (OCD, depression)	Secondary school	N/N	N
13	+	Y, mild	College (assisted)	Y/Y	N

Y; yes, N; no, IQ: intelligence quotient, ADL: activities of daily living, *can walk 400 m without a wheelchair, #: was previously employed, ^: rollator-dependent, + height could not be measured (wheelchair-dependent for many years), weight 45 kg

Four patients had mild cognitive impairment (patient numbers 4, 5, 10 and 13), while cognitive function was normal in the other nine patients (Table 2). Ten patients were employed at any time during their adult life. Pregnancies with healthy offspring were reported in two patients (patient numbers 3 and 11).

### Skeletal pathology

**Fig. 1:**
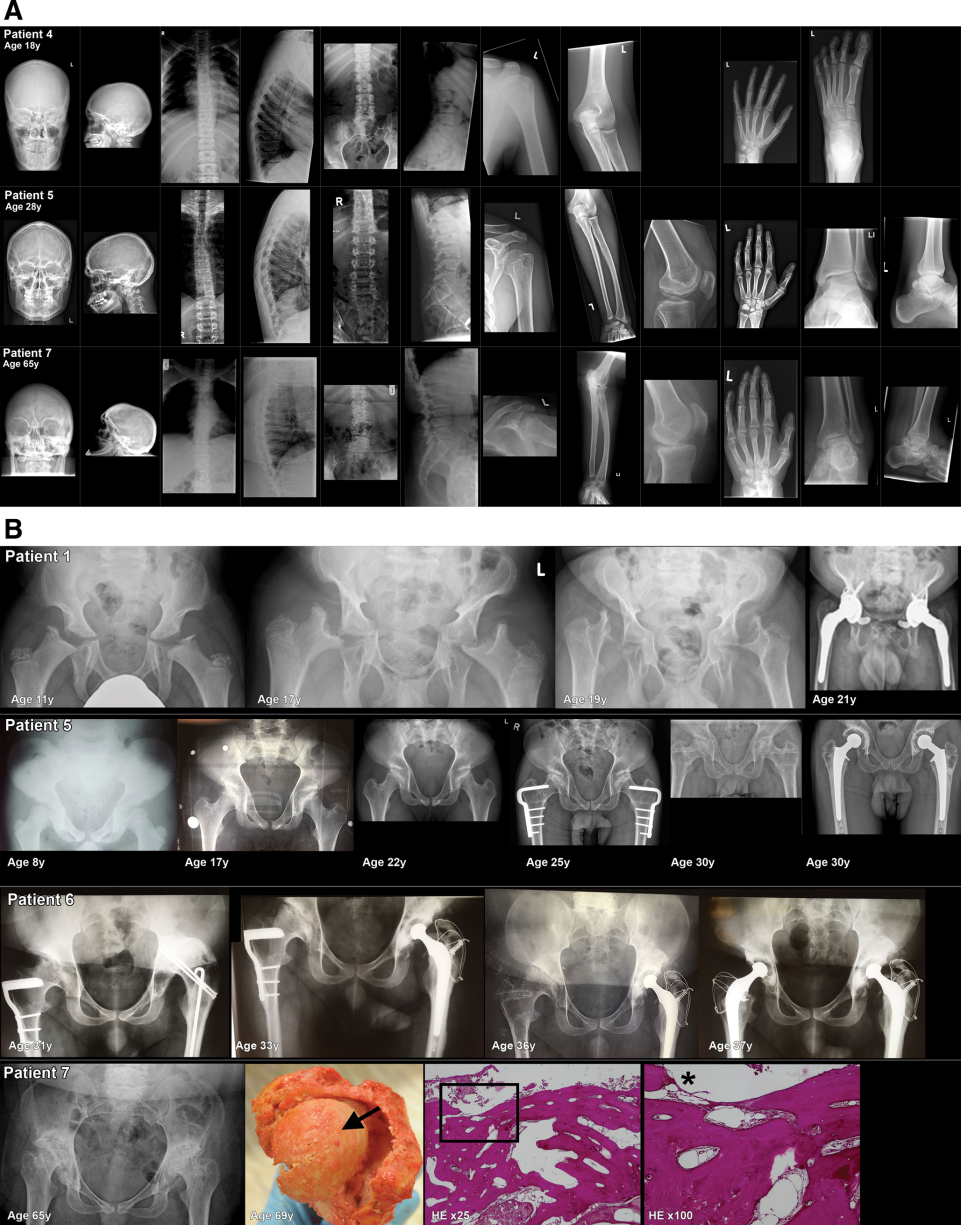
Skeletal radiographs. **a** Examples of three MLIII patients, aged 18, 28 and 65 years (patient numbers 4, 5, and 7). Skeletal radiographs of the skull (anterior posterior and lateral), spine (thoracic/lumbar vertebrae AP and lateral), left shoulder (AP), left elbow (lateral), left knee (AP), left hand (AP) and left ankle/foot (AP and or lateral). In general, the developmental bone abnormalities were present in all patients, but the presence and severity of osteoarthritic changes were more prominent in the older patients. **Skull**: In all patients, thickened cortical bones and a prominent sella turcica were observed. Open skull sutures in patient numbers 4 and 5. Dens aspect of the skull vault in patient number 7. **Spine**: Mild convex right-sided scoliosis, with increased kyphosis and increased interpedicular distances in all three patients. Flattened corpora vertebrae on several levels (cervical, thoracic and lumbar) in all three patients. Osteoarthritic changes of the endplates of the corpus vertebrae, most prominent in the oldest patient (patient number 7). In patient 7, there is anterior displacement of vertebrae L3 and L4 with a decreased diameter of the spinal cannel. **Shoulder and elbows**: In patient number 4, no abnormalities of these joints were observed. Osteoarthritic changes in the humeral head, glenoid and elbow deformation were seen in patient numbers 5 and 7. **Knee**: From patient number 4, no lateral left knee radiograph was available. In patient number 5, there is a patella baja and signs of osteochondral abnormalities of the patella with osteophyte formation. In patient number 7 (X-ray performed at age 60 years), osteoarthritic changes were observed with lateral hook formation/bone formation of the tibia plateau and at the lateral femur condyle. **Hand**: Abnormal shaped phalanges in all three patients (subtle in patient number 4). Osteoarthritic changes of the phalangeal joints (proximal and distal) in patient numbers 5 and 7. Abnormal shaped metacarpal bones (hypoplasia and collapse) with secondary osteoarthritis (patient numbers 5 and 7). **Ankles/feet**: In patient number 4, no abnormalities of the joints were observed. In patient number 5, there is osteoarthritis of the distal fibula. Suggestion for bifida talus or talus bipartite. Severe osteoarthritis of the ankle is seen in patient number 7. **b** Radiographs, macroscopy and histopathology of the hip bones. X-rays of the hips of patient numbers 1, 5, 6 and 7 over different ages. Macro- and microscopic photographs of the left hip of patient number 7 are shown. This patient died at the age of 69 years from metastatic bladder cancer. The most prominent findings on radiographs: **Pelvis**: In all patients, the pelvic bones are abnormally shaped, with flared iliac wings with hypoplasia of the inferior part of the ilea. The acetabula are severe dysplastic, very steep and shallow. Neoacetabulum formation occurred in patient numbers 5, 6 and 7. Impingement of the coxofemoral spaces was seen in patient number 7. **Femoral heads, neck shaft angle**: Severe ossification disorders and severe secondary osteoarthritis of the femoral heads (with subchondral cysts, sclerosis and flattening in patient numbers 5, 6 and 7) were present in all patients. In patient number 1, at age 11 years, there was near total absence of the femoral heads. Femoral shaft angle abnormalities; in patient number 1, the shaft angle over time develops from coxa valga to coxa vara. In patient number 5, there is a coxa valga and in patient number 7 coxa vara. On autopsy in patient number 7, part of the left femur, femoral head and part of the acetabulum were removed, shown on the macroscopic photo. The femoral head (shown from above); severe osteoarthritis is present, with complete destruction of the cartilage. An arrow on the top of the femoral head shows yellow coloured bone tissue and not the normal glossy blue-white in appearance cartilage. Total destruction of cartilage is also illustrated by the histological slides of the upper part of the femoral head coloured with HE, magnification ×25 and ×100. A square on the ×25 magnification indicates the location of the ×100 magnification. No cartilage remains at the location of the asterisk on the ×100 magnification. **Surgical interventions of the hip**: Patient number 1: custom-made total hip replacement (THR) of the left and right hips at 20 and 21 years, respectively. Patient number 5: femoral varus osteotomy at ages 22 years (left hip) and 24 years (right hip) and THR at age 30 years. Patient number 6: femoral varus osteotomy at ages 22 years (left hip) and 24 years (right hip) and THR at 31 years (left hip) and 37 years (right hip)

The most prominent clinical signs were the skeletal abnormalities. All patients had abnormally shaped bones (dysostosis multiplex) and progressive osteoarthritis, characterised by cartilage destruction in joints and areas of radiolucency in bones that may reflect erosive bone lesions (Supplemental Table 1). These abnormalities were found on X-rays of the hand, feet, shoulders, elbows, hips, knees and spine (Freisinger et al. 1992; Hetherington et al. 1999; Haddad et al. 2000; Robinson et al. 2002; David-Vizcarra et al. 2010; Pantoja Zarza and Diez Morrondo 2014; Kadar et al. 2017). In four patients, the carpal and/or tarsal bones were hypoplastic, with secondary osteoarthritic changes observed in the older patients (examples in Fig. 1a and Supplemental Table 1). In 11 patients, the same abnormalities were seen in the humeral/ femoral heads and femoral neck (examples in Fig. 1a, b). In all patients of whom data on hip morphology were available (*n* = 9), hip dysplasia and altered pelvic shape were present (examples in Fig. 1b). Abnormalities of the spine were present in all patients; the most common findings were atypically shaped vertebrae (hypoplasia), subluxation and scoliosis (examples in Fig. 1a). The majority of patients reported pain of the glenohumeral joints, and/or the hands, feet, hips, knees and the lumbar spine. In six patients, signs of spinal cord or nerve root compression were present (Supplemental Table 1).

Figure 1a shows exemplary radiographs of the skull, spine, shoulder, elbow, knee, hand, ankle and foot of three MLIII patients (patient numbers 4, 5 and 7) at the ages of 18, 28 and 65 years, respectively. The findings in these radiographs are described in the legend of Fig. 1a. Separately, X-rays of the pelvis of four MLIII patients (patient numbers 1, 5, 6 and 7) over time are shown in Fig. 1b. In all patients, there is severe hip dysplasia, with flaring of iliac wings as well as significant ossification disorders of the femoral heads, with arthritic changes of the hips. In the most severely affected patient (patient number 1), there is near total destruction of the femoral heads by the age of 11 years. In contrast, in patient number 5, the femoral heads are hardly affected at the age of 8 years, but by the second decade of life, severe osteoarthritis of both hips had developed.

The oldest patient (patient number 7) was completely wheelchair-dependent from the age of 23 years. In this patient, no surgical hip interventions have been performed, for unknown reasons. The femoral heads were abnormally shaped, with severe secondary osteoarthritis. This is also seen on macroscopic and histopathological examination of the left hip, which was removed after her death at the age of 69 years (Fig. 1b), when she succumbed to metastatic bladder cancer.

### Orthopaedic surgical interventions and medical treatment

**Fig. 2:**
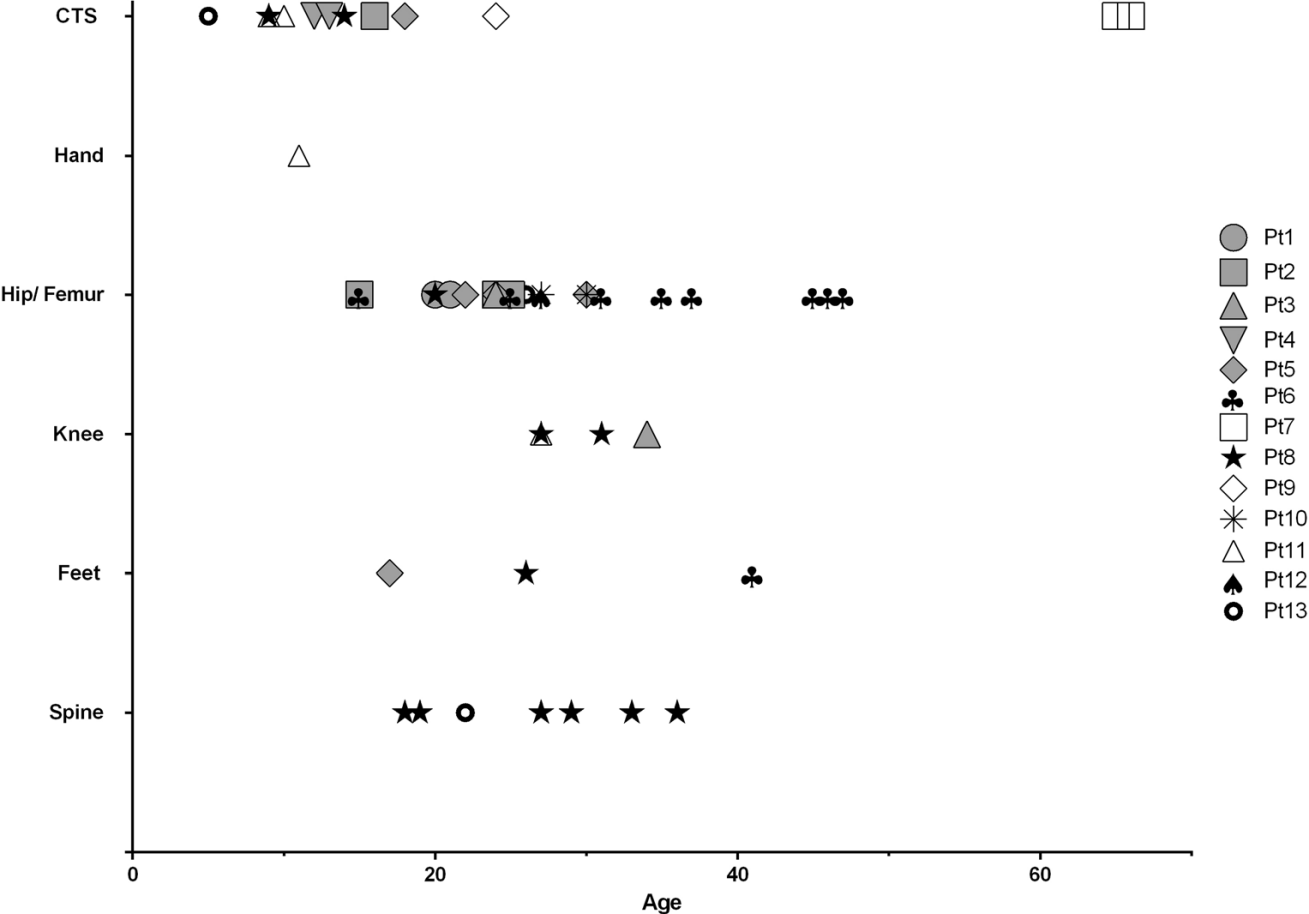
Orthopaedic surgical interventions. All orthopaedic surgical interventions of 13 MLIII patients and the age at which they were performed are shown. In some patients, the same surgical intervention was performed more than once

For all patients, the type of orthopaedic surgeries and age at which these were performed are depicted in Fig. 2. Details of the specific surgical procedures can be found in Supplemental Table 2. The most frequent intervention was hip surgery, performed in eight patients, with first interventions in the second or third decades of life. In all of these patients, total hip replacement (THR) was eventually required.

Eight patients underwent bilateral CTS release, mostly in the second decade of life. In one patient, this intervention was performed more than once. Less frequently, surgical interventions of the knees, feet and spinal cord were performed. Several patients were treated with repeated corticosteroids injections in the glenohumeral or knee joint to reduce pain. Two patients were treated with bisphosphonates at their last outpatient visit (patient numbers 8 and 10).

### Cardiac and pulmonary examinations

Echocardiography was performed in 12 patients (Supplemental Table 3). In patient number 4, there were limited signs of cardiac hypertrophy, with thickening of the posterior left ventricular wall. Systolic ventricular function was normal in all patients. Mild dilated right ventricle with normal systolic function was seen in patient number 10. Five patients (patient numbers 1, 4, 5, 10 and 13) had mild regurgitation of one or more valves (mitral, aortic, pulmonary or tricuspid) and one patient had moderate stenosis and regurgitation of the aortic valve (patient number 5). None of the patients had required valve replacement at the time of their last follow-up.

Pulmonary function tests were performed in six patients (Supplemental Table 3). Two patients had mild to moderate restrictive lung disease (patient numbers 1 and 4).

## Discussion

This multi-centre retrospective medical record review describes the clinical course of adult forms of MLIII. About half of the patients experienced significant physical limitations, being either wheelchair-dependent and/or needing help with ADL. Pain was reported by all patients. Approximately one-third of the patients had mild cognitive impairment. Three-fourths of patients had been employed at any time during adulthood.

All patients have extensive skeletal pathology, requiring orthopaedic surgical interventions as early as the second or third decades of life. THR was the most common intervention, performed in 67% of all patients. In three patients, this was preceded by femoral varus osteotomy, but despite this position correction, these patients still needed THR some 10 years later (Fig. 1b, Supplemental Table 2).

As is seen in the different forms of MPS, abnormal bone development (dysostosis multiplex) (Maroteaux and Lamy 1966) is uniformly present in MLIII patients. Clinically, the earliest disabling feature is hip disease, characterised by pain and limited mobility. Abnormal hip morphology (acetabula, iliac bones and femoral heads) has been observed in very young MLIII patients [at birth and at ages 4 and 6 years (Hetherington et al. 1999; Cathey et al. 2010; David-Vizcarra et al. 2010)], suggesting early developmental alterations such as seen in, for example, MPS VI (Oussoren et al. 2017).

In addition to the dysostosis multiplex, throughout life, the joints in MLIII are affected by rapidly progressive osteoarthritis, resulting in cartilage destruction and bone lesions (areas of radiolucency and sclerosis). Clinically, all patients suffer from bone and joint pain.

Bone disease in MLIII may arise from an imbalance between bone-forming osteoblasts and bone-resorbing osteoclasts, caused by the increased presence of osteoclastic enzymes in the bone-resorbing zone in osteoclasts (Kollmann et al. 2013). Mannose-6-phosphate is important for the trafficking of these enzymes along the exocytic pathway to the apical membrane, where they are secreted in the bone-resorbing compartment (Baron et al. 1988). The absence of mannose-6-phosphate on the osteoclastic enzymes may lead to increased secretion of these enzymes, resulting in uncontrolled bone and cartilage degradation (Barriocanal et al. 1986; Robinson et al. 2002). However, this hypothesis still needs to be substantiated by pathophysiological studies.

CTS was highly prevalent in our MLIII patient population (11 out of 13 patients) and bilateral CTS release was performed in eight patients. Cardiac valve abnormalities were found in six patients; there were no signs of cardiac dysfunction. Six patients underwent formal pulmonary function assessment and two patients had moderate to mild restriction, most likely due to thoracic skeletal abnormalities. No remarks concerning airway infections or pulmonary complaints were present in the medical records. This distinguishes this condition from the different forms of MPS (e.g. types I, II, IV and VI), in which cardiac and pulmonary problems are more frequent, often with severe clinical implications, also in the milder adult forms of these disorders (Brands et al. 2013; Clark et al. 2017; Rapoport and Mitchell 2017).

### Future therapies

Future therapies for MLIII should aim to improve bone metabolism, in order to reduce bone pain, delay the need for surgical intervention and improve mobility. Bisphosphonates are given in MLIII patients to decrease osteoclastic activity, with variable outcomes (Robinson et al. 2002; Zolkipli et al. 2005; Kerr et al. 2011; Kobayashi et al. 2011). Since the long-term use of these drugs suppresses bone turnover and may have a negative effect on length growth, they may be of limited use in MLIII. A newer anti-bone resorption drug, Denosumab (blocking the osteoclast activating cytokine receptor activator of NFκB ligand), may hold promise for the treatment of ML and has already been used with some success in osteogenesis imperfecta, improving both growth and vertebral shape (Hanley et al. 2012, 2017; Shaker et al. 2015). Another option for treatment may be reduction of inflammation, by drugs such as pentosan polysulfate (PPS), which has been shown to improve range of motion and reduce pain in MPS I patients (Hennermann et al. 2016). Future pathophysiological studies on the characteristics of bone metabolism in MLIII will be needed in order to establish the most promising therapeutic option in this disease.

## Conclusion

Severe skeletal abnormalities, resulting from abnormal bone development and severe progressive osteoarthritis, are the hallmark of mucolipidosis type III (MLIII), necessitating surgical orthopaedic interventions early in life. Future therapies for this disease should focus on improving cartilage and bone quality, preventing skeletal complications and improving mobility.

## Electronic supplementary material

Below are the links to the electronic supplementary material.

ESM 1 (DOCX 21 kb)

ESM 2 (DOCX 15 kb)

ESM 3 (DOCX 14 kb)
