# Supplementary material for: Mucolipidosis type III, a series of adult patients
Source: J Inherit Metab Dis. 2018 Apr 27;41(5):839–48. doi: 10.1007/s10545-018-0186-z (PMC6133174; doi:10.1007/s10545-018-0186-z)
Supplement: Supplementary file 1 — (DOCX 21 kb) [file 10545_2018_186_MOESM1_ESM.docx]

| **Supplemental Table 1 :** **Skeletal abnormalities** | | | | | |
| --- | --- | --- | --- | --- | --- |
| **Patient** | **Shoulders and Hands** | **Pelvis** | **Femur** | **Knees and feet** | **Spine, spinal cord compression** |
| 1 |  | Severe hip dysplasia. Os ilium: neo-acetabulum formation and flaring wings  (19 yrs) | Dislocation/luxation femur to cranial in relation to the acetabulum. Absence of femoral heads. Shaft: osteopenia  (19 yrs) |  | Flattened vertebral bodies, hypoplastic dens, cervical pannus around dens-atlas area, thoracal, lumbar platylospondyli.  Craniocervical compression spinal cord  (20 yrs) |
| 2 |  | Acetabula steep, barely covering femoral heads  (24 yrs) | Epiphyseal dysplasia  (6 yrs)  Femoral heads subluxation to cranial L>R, subchondral areas of radiolucency  (24 yrs)  Secondary osteoarthritis femoral heads after femoral osteotomy, L>R  (25 yrs) | Irregular aspect dorsal side patella, slight intra-articular effusion  (24 yrs)  Upper ankle joints early signs of secondary osteoarthritis L/R  (27 yrs) | S shape scoliosis  L1 to L5: secondary osteoarthritis degenerative changes with multiple Schmorl nodules  L2-L3, L3-L4, L4-L5: slight bulging disc  (24 yrs) |
| 3 |  | Flaring os ileum and dysplasia  (42 yrs) |  | Secondary osteoarthritis L knee joint abnormal aspect of the bones, tibiofemoral joint space slightly narrowed medial, areas of radiolucency medial condyle and R tibia  (36 yrs) | Thoracolumbar severe convex left sided scoliosis, osteopenia, secondary osteoarthritis, degeneration L1-L2  Flattened vertebral bodies, mainly cervical increased anteroposterior diameter  Sclerosis SI-joint  (42 yrs) |
| 4 |  | Mild horizontal acetabulum roof R  (18 yrs) | Mild coxa recta L/R  (18 yrs) |  | Mild convex right sided scoliosis, with increased kyphosis and increased interpedicular distance  Secondary osteoarthritic changes of the endplates of the corpus vertebrae  (18 yrs) |
| 5 | Glenoid deformation L/R Madelung’s deformity L/R  (28 yrs)  Caput MCP: deformities, secondary osteoarthritis with collaps and areas of radiolucency  Os lunatum L/R: hypoplasia proximal pool. Os scaphoid L/R: absence proximal pool  (30 yrs) | Hip dysplasia L/R  (28 yrs) | Secondary osteoarthritis femoral heads L/R (28 yrs) | Multiple abnormalities feet R>L (dysostosis multiplex)  (17 yrs) | Mild convex right sided scoliosis, with increased kyphosis and increased interpedicular distance  Secondary osteoarthritis endplates of the corpus vertebrae  (28 yrs) |
| 6 | Glenoids hypoplasia and secondary osteoarthritis  (38 yrs)  Severe secondary osteoarthric changes of the wrist bones and MCPJ L/R:  (44 yrs) | Flaring iliac wings, acetabulum  (45 yrs) | Secondary osteoarthritis femoral heads L/R  (25 yrs) | Osteopenia, secondary osteoarthritis tibiofemoral and patello wih femoral joint space narrowing  Secondary osteoarthritis knees  (50 yrs)  Abnormal talus, tibia and caput MT-I (sclerosis)  Flat caput metatarsalia with secondary osteoarthritis (44 yrs) | Hypoplasia of dens and vertebral bodies C3 to C7  C4-C5 and C5-C6: bilateral narrowing foramen intervertebralis  Spinal cord compression C3 -C5  Radicular syndrome C6 L  (50 yrs) |
| 7 | Severe secondary osteoarthritis humeral heads (subcortical areas of radiolucency, sclerosis)  (65 yrs)  Distal part ulna, os scaphoid, os lunatum, MCPJ-III and IPJ: deformities, destruction, arthritis  (60 yrs) | Hip dysplasia  (60 yrs) | Dysplasia, flattening and cranial subluxations. Severe secondary osteoarthritis (subchondral sclerosis and areas of radiolucency) femoral heads with joint space narrowing  Coxa valga L/R  (60 yrs)  Flattening and lateralization femoral heads Distal femur shaft fracture  (65 yrs) | Knee joint; severe secondary osteoarthritis (narrowing of the medial compartment, deformation of the tibia plateau with lateral hook formation/bone formation)  Secondary osteoarthritis (hook formation at the lateral femur condyles) with deformed aspect femur condyles L/R  (65 yrs) | Altered vertebral shape, flattened (most prominent TH9/TH10). L3 and L4: anterior displacement  Mild convex right sided scoliosis, with increased kyphosis and increased interpedicular distance  Severe secondary osteoarthritis endplates of the corpus vertebrae  L3-L4 and L4-L5: decreased diameter of the spinal cannel  (66 yrs) |
| 8 | Secondary osteoarthritis glenohumeral joint  PIPJ dig II R: secondary osteoarthritic changes  (30 yrs) | Hip dysplasia |  | Avascular necrosis ankles  (10 yrs)  Secondary osteoarthritis with degenerative changes ankles, some collapse talus. Bilateral valgus knee deformities  (25 yrs)  Hammer toe R  (30 yrs)  Ankle secondary osteoarthritis R, predominant anterior wear and slight anterior subluxation  (34 yrs) | Antlantoaxial subluxation os odontoideum  Loss of cervical lordosis  C2, C3: grade 2 Enterolisthesis  C4 - C5 and C5 - C6: Grade 1 rethrolisthesis  Multiple osteophyte bars  C4 to C7: several multilevel bilateral neural foraminal narrowing with indention of the cervical cord  L5: spinal cord compression resulting in weakness of the right side  (30 yrs)  T10/T11: severe stenosis resulting in clonus on leg L/R  (34 yrs) |
| 9 |  | Unknown |  |  | Cervical spine loss of normal lordosis and secondary osteoarthritic changes; degenerative change of the intervertebral discs, cervical, thoracic, lumbar regions  Some narrowing of the neural exit foramina at C2-3, C3-4 bilaterally  At the cranial cervical junction there is a Chiari I malformation with the right cerebellar tonsil extending inferiorly to the lower edge of the C1 arch  (23 yrs) |
| 10 |  | Unknown | Secondary osteoarthritic changes femoral head L  (35 yrs) |  | Secondary osteoarthritic degenerative changes whole spinal cord. Decreased diameter of the spinal cannel at multiple levels  (30 yrs)  Severe marked cervical spondylosis, multiple disc bulges  Some nerve root compression  (34 yrs) |
| 11 | IPJ: fixed flexion and deformities, secondary osteoarthritis |  |  | Loose fragments in the articular surfaces of patellae (associated with fissuring and chondral flaps) L/R  (32 yrs) | Mild convex right site lumbar scoliosis  C2-C3: grade 1 anterolisthesis  (30 yrs)  C6-7 (and to a lesser degree C7-T1): secondary osteoarthritis (degenerative bone and disc changes)  C5-6: modest degree of foraminal compromise  (32 yrs) |
| 12 |  | Hip joint L/R: Loss of height, secondary osteoarthritis (subchondral sclerosis, changes acetabular margins)  (24 yrs) | Sclerosis, osteophytes, progressive secondary osteoarthritis femoral heads L/R  (24 yrs)  Extensive secondary osteoarthritis femoral heads L/R  (26 yrs) | Hallux valgus with deformed hammer toes L  (29 yrs) | Intractable right sided L5 radiculopathy and is stuck in forward flexion  Cervical, thoracic and lumbar spine: multilevel secondary osteoarthritis (degenerative disc changes)  (30 yrs) |
| 13 | Hypoplastic poorly formed glenoid fossae  Varus positioning of both humeral heads  (22 yrs) | Very shallow acetabuli  Incomplete fusion of right pubic ramus  Widening and erosion of joints and growth plates  (18 yrs) | Flat and deformed femoral heads  (18 yrs) |  | Loss of vertebral body height ad end-plate changes throughout  Severe narrowing of and compression of spinal cord at cranio-cervical junction (pre-laminoplasty)  (19 yrs) |
| MCP: metacarpal, L: left, R: Right, yrs=years, SI-joint: sacroiliac joint, MCPJ: metacarpal joint, IPJ: Interphalangeal joint, PIPJ: proximal interphalangeal joint, Dig: digitus, THR: total hip replacement, MT: metatarsals.  For each finding, the age at which this was written down in the medical chart is depicted, this does not necesserily correspond with the age of onset of the symptom or sign. If no age is given, the symptom or sign was noted in the chart undated. | | | | | |
