# Supplementary material for: Mucolipidosis type III, a series of adult patients
Source: J Inherit Metab Dis. 2018 Apr 27;41(5):839–48. doi: 10.1007/s10545-018-0186-z (PMC6133174; doi:10.1007/s10545-018-0186-z)
Supplement: Supplementary file 2 — (DOCX 15 kb) [file 10545_2018_186_MOESM2_ESM.docx]

| **Supplemental table 2: Orthopedic surgeries** | | | | | | | | |
| --- | --- | --- | --- | --- | --- | --- | --- | --- |
| **Patient** | **CTS**  **Age (years)** | | **Pelvis, Femur**  **Age (years)** | | **Hands, knees and feet**  **Age (Years)** | | **Spine, spinal cord**  **Age (Years)** | |
| **1** |  |  | 20& 21 | Custom-made THR L, THR R with distal tenotomy hamstring R |  |  |  |  |
| **2** | 16 | CTS release L/R | 15  24& 25 | Femoral varus osteotomy and endorotating osteotomy proximal femur L/R  THR (sequential) L/R |  |  |  |  |
| **3** | # |  | 24 | THR (sequential) L/R | 34 | Total knee prosthesis R |  |  |
| **4** | 12&13 | CTS release R/L |  |  |  |  |  |  |
| **5** | 18 | CTS release L/R | 22& 24  30 | Femoral varus osteotomy (sequential) L/R  THR (sequential) R/L | 17 | Bunioectomy metatarsalia,  PIP resection dig. V L/R feet |  |  |
| **6** |  |  | 15  25  31  35  37  45  45  46&47  47  47 | Femoral varus osteotomy R  Arthrodesis L hip  THR L (Charnley)  Removal of bridge plate R  THR trochanterosteotomy R  Osteosynthesis femur R (femur fracture R)  Bridge plate failure, reposition, fixation fracture distal femur R with plate osteosynthesis  Re-osteosynthesis femur R, allograft bone grafting  Drainage abscess (R femur)  Removing osteosynthesis material, resection pseudarthrosis part R femur, placement angle blade plate R femur condyl | 41 | Arthrodesis upper ankle joint |  |  |
| **7** | 65&66 | CTS release L/R |  |  |  |  |  |  |
| **8** | 9-14 | Multiple CTS releases | 20 | THR (sequential) L/R | 26  27  31 | Arthroscopic debridement ankle R  Arthroscopy knee L  Total knee replacement L | 18  19  27  29  29  33  36 | C1/C2 fusion secondary to cervical fracture  Transarticular screws os odontoidenum  L4/L5 and sacral fusion  L4-S2 anterior fusion  C4/5, C5/6, C6/7 bilateral foraminoplasties  T10-11 post. decompressive laminectomy, extension of the posterior fixation to T10  Occipito-cervical-thoracic fusion |
| **9** | 24 | CTS release L/R |  |  |  |  |  |  |
| **10** | # |  | 27& 30 | Uncemented THR (sequential) R/ L |  |  |  |  |
| **11** | 9&10 | CTS release L/R |  |  | 11  27 | Surgery for trigger fingers, tendon release bilaterally (ring finger)  Knee arthroscopy and washout L/R |  |  |
| **12** | # |  | 27 | Uncemented Ceramic THR (subsequential) L/R |  |  |  |  |
| **13** | 5 | Bilateral CTS release | 26 | Excision arthroplasty of R hip |  |  | 22 | C3-T1 laminoplasty |
| CTS; carpal tunnel syndrome, # CTS present bilateral, no surgical intervention, THR; total hip replacement, L: left, R: right; PIP; proximal interphalangeal, dig; digits | | | | | | | | |
