# Supplementary material for: Mucolipidosis type III, a series of adult patients
Source: J Inherit Metab Dis. 2018 Apr 27;41(5):839–48. doi: 10.1007/s10545-018-0186-z (PMC6133174; doi:10.1007/s10545-018-0186-z)
Supplement: Supplementary file 3 — (DOCX 14 kb) [file 10545_2018_186_MOESM3_ESM.docx]

| **Supplemental Table 3:** Cardiac and pulmonary evaluations | | | | | | | | | |
| --- | --- | --- | --- | --- | --- | --- | --- | --- | --- |
| 1. **Cardiac evaluation** | | | | | | | | | |
| **Patient** | | **Age cardiac evaluation (years)** | | **LVH** | | **LV function** | | **Other abnormalities** | |
| 1 | | 20 | | No | | Normal | | Mild AR and MR | |
| 2 | | 21 | | No | | Normal | | No | |
| 3 | | 43 | | No | | Normal | | Myxomatous mitral valve with redundant tissue, minor billowing, no insufficiency | |
| 4 | | 16 | | No | | Normal | | Mild MR. Posterior left ventricular wall slightly thickened | |
| 5 | | 29 | | No | | Normal | | Bicuspid aortic valve, mild AS, moderate AR, mild MR and TR | |
| 6 | | N.A. | | N.A. | | N.A. | | N.A. | |
| 7 | | 65 | | No | | Normal | | No | |
| 8 | | 32 | | No | | Normal | | No | |
| 9 | | 28 | | No | | Normal | | No | |
| 10 | | 35 | | No | | Normal | | Mild/ moderate TR. Mildly dilated RV with normal systolic function | |
| 11 | | 30 | | No | | Normal | | No | |
| 12 | | 35 | | No | | Normal | | No | |
| 13 | | 26 | | No | | Normal | | Moderate/ severe AR, mild MR, PR and TR | |
| 1. **Pulmonary evaluation** | | | | | | | | | |
| **Patient** | **Age at spirometry (years)** | | **FVC (% predicted)** | | **FEV1 (% predicted)** | | **FEV1/VC (% predicted)** | | **Comments** |
| 1 | 23 | | 60 | | 52 | | 96 | | Moderate restrictive spirometry without obstructive characteristics |
| 3 | 46 | | 116 | | 121 | | 105 | | No restriction, no obstruction |
| 4 | 17 | | 74 | | 78 | | 78 | | Mild Restriction |
| 5 | 30 | | 101 | | 85 | | 85 | |  |
| 7 | 66 | | 89 | | 88 | | 72 | | No airway restriction or obstruction, normal flow-volume curve |
| 13 | 24 | | 36 | | 37 | | 105 | |  |
| N.A.: not available, LVH: left ventricle hypertrophy; LV: left ventricle; RV: right ventricle; N.A.: not available, AR: aortic regurgitation, MR: mitral regurgitation, AS: aortic stenosis, TR: tricuspid regurgitation, PR: pulmonary regurgitation, FVC: forced vital capacity; FEV1: Forced expiratory volume in 1 second; VC: vital capacity | | | | | | | | | |
